# 6-Thioguanine is a noncompetitive and slow binding inhibitor of human deubiquitinating protease USP2

**DOI:** 10.1038/s41598-018-21476-w

**Published:** 2018-02-15

**Authors:** Shang-Ju Chuang, Shu-Chun Cheng, Hui-Chi Tang, Chiao-Yin Sun, Chi-Yuan Chou

**Affiliations:** 1 Department of Life Sciences and Institute of Genome Sciences, National Yang-Ming University, Taipei, 112 Taiwan; 2 Department of Nephrology, Chang-Gung Memorial Hospital, Keelung, 204 Taiwan

## Abstract

Ubiquitin-specific protease 2 (USP2) belongs to the family of deubiquitinases that can rescue protein targets from proteasomal degradation by reversing their ubiquitination. In various cancers, including prostate cancer and ovarian carcinoma, upregulation of USP2 leads to an increase in the levels of deubiquitinated substrates such as fatty acid synthase, MDM2, cyclin D1 and Aurora-A. USP2 thus plays a critical role in tumor cells’ survival and therefore represents a therapeutic target. Here a leukemia drug, 6-thioguanine, was found to be a potent inhibitor of USP2. Enzyme-kinetic and X-ray crystallographic data suggest that 6-thioguanine displays a noncompetitive and slow-binding inhibitory mechanism against USP2. Our study provides a clear rationale for the clinical evaluation of 6-thioguanine for USP2-upregulated cancers.

## Introduction

Ubiquitination, a kind of post-translational modification, as well as deubiquitination, which together comprise the ubiquitination system, can regulate not only the turnover of cellular proteins but also, through various modes of mono- or polyubiquitination, receptor trafficking, DNA repair, cell-cycle progression, immune response, autophagy and apoptosis^1^. This versatility has raised interest in pinpointing components of the ubiquitin system as prime candidates for drug targets^2^. Among them, the pursuit of deubiquitinases (DUBs) as pharmaceutical targets is still in its infancy in that few inhibitors have entered clinical trials, albeit some broad-spectrum inhibitors have been patented and highly selective inhibitors have been developed^3–6^. The human genome encodes about 80–90 DUBs, of which the largest of the five DUB families is the ubiquitin-specific proteases (USPs)^7^. One of these, USP2, is able to remove ubiquitin from a range of protein substrates such as fatty acid synthase^8^, MDM2^9^, cyclin D1^10^ and Aurora-A^11^. Fatty acid synthase has been reported to be highly expressed in a variety of cancers, including cancers of the prostate, ovary, lung, colon, and breast^12^, while MDM2, cyclin D1 and Aurora-A play critical roles in p53 regulation, cell cycle progression and mitotic events, respectively^9,10,13^. All of these studies suggest that the inhibition of USP2 may be a good therapeutic approach against cancers. In 2016, Davis *et al*.^6^ reported that a small molecule, ML364, can induce cell cycle arrest and inhibit the growth of colorectal and mantle cell lymphoma cell lines via USP2 inhibition, albeit it is still not a clinical drug. In the present study, 6-thioguanine (6TG), an old drug still clinically used to treat leukemia, rheumatoid arthritis and bowel disease^14^, was identified as a potent USP2 inhibitor. Further studies using enzyme kinetics and X-ray crystallography to delineate its direct binding to USP2 were performed. Our findings support earlier results suggesting that 6TG has a broad spectrum of activity^15–17^, and have implications with respect to the need for direct clinical evaluation of its use against USP2-upregulated cancers.

## Results and Discussion

### Identification of inhibitors of human USP2

**Figure 1:**
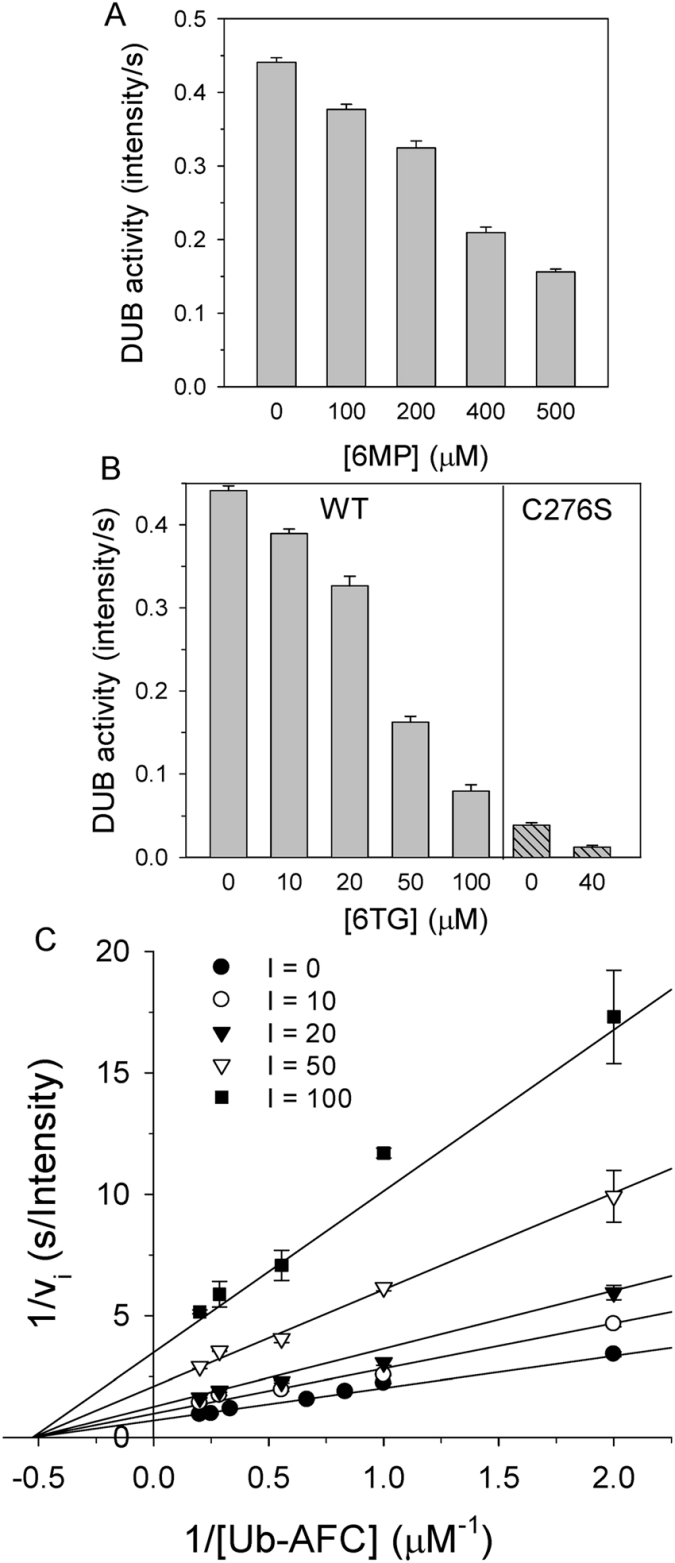
Inhibition of human USP2 deubiquitinating activity by 6MP or 6TG. (**A**) The activity of USP2 in the presence of various concentrations of 6MP was measured. (**B**) The activity of USP2 (left panel) or its C276S mutant (right panel) in the presence of various concentrations of 6TG was measured. In panel A and B, 1 μM Ub-AFC and 0.2 μM USP2 was used as the substrate and the enzyme, respectively. (**C**) Noncompetitive inhibition of USP2 by 6TG. Enzyme activity was measured under various concentrations of Ub-AFC (0.5–5 μM) and 6TG (0–100 μM), while protein concentration was held at 0.2 μM. The assays were performed in 20 mM phosphate (pH 7.6) at 30 °C and repeated to ensure reproducibility. The symbols and bars represent the mean and standard error, respectively. The solid lines represent the best global fit to the noncompetitive inhibition equation. R_sqr_ is 0.981 and the kinetic parameters from the best fit are shown in Table 1.

According to previous studies involving molecular docking experiments, the anti-leukemia and immunosuppressant drugs 6MP and 6TG, which act as inhibitors of SARS and MERS coronavirus papain-like proteases (PLpros), may also be able to bind to the active site of human USP2 as both kinds of enzymes share the palm-thumb-fingers structural scaffold and conserved catalytic triad Cys-His-Asp/Asn^17–20^. To validate the simulation results, the deubiquitination activity of the recombinant human USP2 catalytic domain (residues 258–605) was measured in the presence of various concentrations of 6MP or 6TG (Fig. 1A,B and Table 1). Interestingly, only 6TG but not 6MP shows a significant inhibitory effect with an IC_50_ of 40 ± 2.0 μM (Fig. 1A,B), whereas both compounds can inhibit coronaviral PLpro with an IC_50_ of 25 μM^17,18^. It is the first demonstration that besides serving as an anti-leukemia drug, 6TG might also be an effective chemotherapeutic agent against cancers dependent on USP2 upregulation^8,21,22^. Similar concept has been addressed by Davis *et al*.^6^. Using the strategies of high-throughput screening and chemical medicinal optimization, they identified a sulfamidobenzamide derivative, ML364, having an IC_50_ of 1.1 μM against USP2 (Table 1) and resulting in the growth arrest of several kinds of USP2-upregulated cancer cells. However, it is not a clinical medicine yet and the detailed inhibition mechanism is still unclear.

**Table 1:** Kinetic parameters of 6MP and 6TG inhibition of human USP2 via deubiquitination assay.

USP2 in	Structure	K_is_ (μM)	K_inact_ (μM)^b^
6 MP^a^		349.0 ± 23.7	—
6TG^a^		24.6 ± 1.8	15.6 ± 3.2
ML364^c^		IC_50_: 1.1 μM	

^a^In the presence of 6MP or 6TG, the apparent kinetic parameters were determined from the best fit of the data to a noncompetitive inhibition model (Eq. 1). The R_sqr_ were 0.980 and 0.981 for that of 6MP and 6TG, respectively.

^b^The K_inact_ was determined from the best fit of the data to the saturation equation.

^c^ML364 is a USP2 inhibitor and the IC_50_ value was from Davis *et al*.^6^.

### Inhibition mechanism of 6TG

To understand the detailed kinetic mechanisms involved in the interaction of 6TG with USP2, the deubiquitination activity of the enzyme was measured under various combinations of inhibitor concentrations and Ub-AFC substrate concentrations. The inhibition data were globally fitted to all possible kinetic models including competitive, noncompetitive, uncompetitive and mixed inhibition models. Interestingly, the results suggest that a noncompetitive inhibition pattern can best describe the inhibition by 6TG with the double reciprocal plot showing all lines intercepting the x-axis and a K_is_ of 24.6 μM (Fig. 1C and Table 1). On the other hand, although 6MP also showed a noncompetitive inhibition pattern, the K_is_ of 6MP inhibition is 14-fold higher than that of 6TG (Table 1). The K_is_ for 6TG inhibition was 11-fold higher than the K_M_ (2.3 ± 0.2 μM) of the deubiquitination reaction, whereas that for 6MP inhibition was 152-fold higher than the K_M_ of the deubiquitination reaction. The latter K_is_ value indicates the ineffectiveness of 6MP for USP2 inhibition. In contrast, both 6TG and 6MP show a competitive inhibition pattern against coronaviral PLpro^17,18^.

### X-ray structure determination of human USP2 catalytic domain in complex with Ub and 6TG

**Figure 2:**
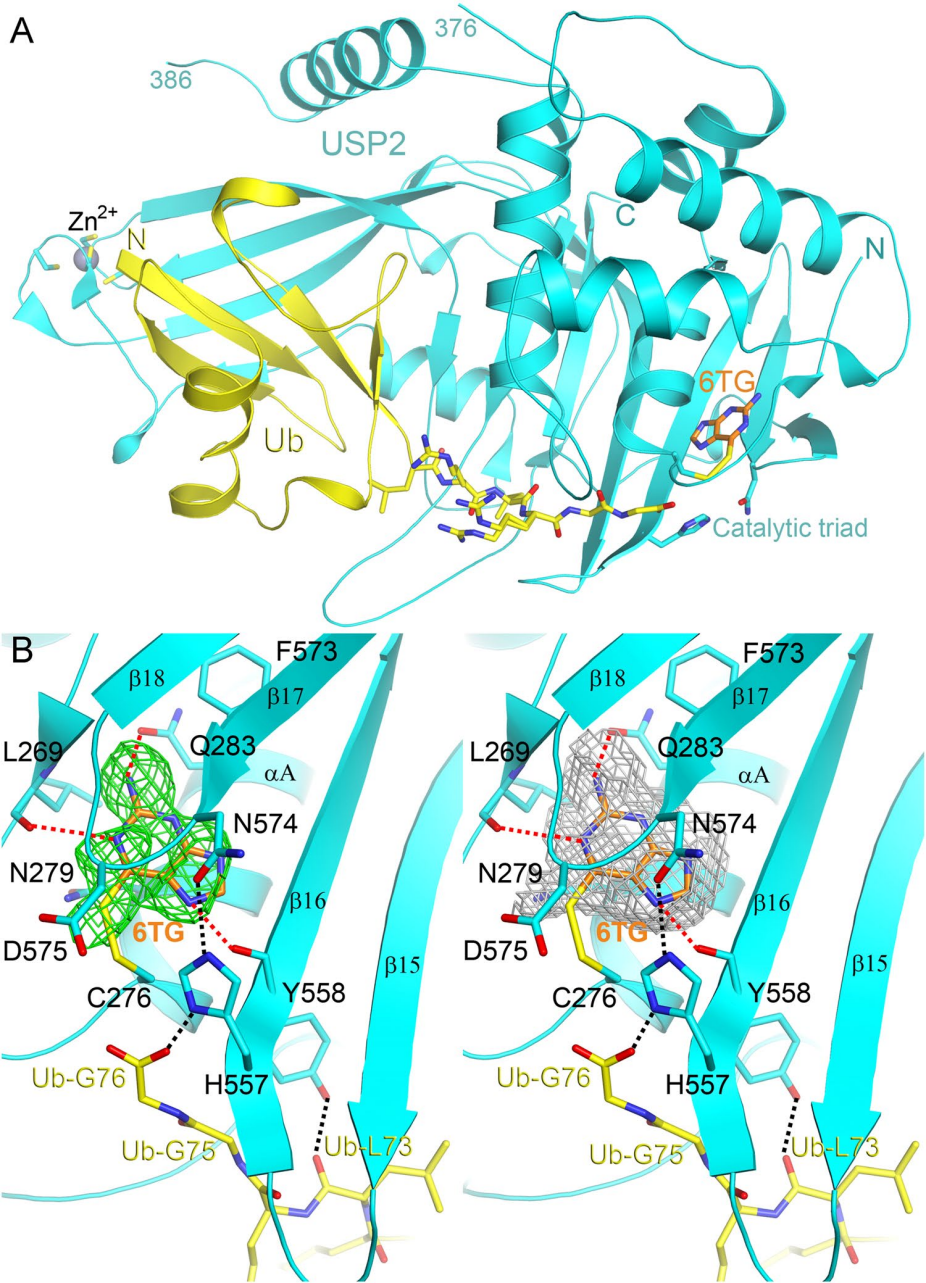
Structure of human USP2 in complex with Ub and 6TG. (**A**) The overall structure of the USP2 258–605 fragment (cyan) in complex with ubiquitin (yellow) and 6TG (orange). In the structure, the residues from 377 to 385 (dashed line) are missing. The zinc ion, coordinated by four cysteine residues, is shown as a grey sphere while the catalytic triad (Cys276-His557-Asn574) and the final six C-terminal residues of Ub are shown as sticks. (**B**) Stereo view of the active site of USP2. The omit *F*_*o*_–*F*_*c*_ electron-density map of 6TG at 1.8 Å, contoured at 2.5σ, is shown as green mesh. To improve the OMIT map quality, the polder map of 6TG (grey mesh) excluding bulk solvent contoured at 1.2σ was generated using PHENIX^31,32^. The dashed lines indicate hydrophilic interactions between USP2 and 6TG or Ub. One of the catalytic triad residues, Cys276, has a disulfide bonding interaction with 6TG. All structure figures in this paper were produced using PyMol (http://www.pymol.org/).

Up to now, although USP2 inhibitors such as ML364 and chalcone-based small molecules^5,6^ have been discovered, no USP2-inhibitor complex structure has yet been solved. Moreover, as a noncompetitive inhibitor may show benefit in combination or adjuvant treatment with a competitive inhibitor, we are very interested in validating the binding mode of 6TG to USP2. Therefore, we tried to determine the structure of USP2 in complex with 6TG by X-ray crystallography. Although repeated attempts at crystallization of USP2 in complex with 6TG failed, we finally succeeded in the crystallization of the USP2 in complex with Ub and then soaked the crystals in the presence of 6TG. The crystal structure of the USP2-Ub-6TG complex was determined at 1.8 Å resolution (Table 2 and Fig. 2A). Fortunately, some residual electron density like 6TG molecule lied on one side of the catalytic triad of USP2, while Ub is located on the opposite side (Fig. 2B and extended data Fig. 1). There is no residual electron density like 6TG in the same place while refined using the diffraction data without 6TG soaking. However, after refinement, the temperature factor of 6TG is 65.2 Å^2^, higher than 38.9 Å^2^ of the protein. Decrease the occupancy to 50% can lower the temperature factor of the molecule to 40.9 Å^2^. It indicates that 6TG may not exist as a full but half occupancy in an asymmetric unit. We have tried to increase the soaking concentration or soaking time to enhance the occupancy or electron density but failed. The co-crystallization of the protein with 6TG also failed. Nevertheless, the structure indicates that 6TG has polar interactions with residues Leu269, Gln283 and Tyr558 and van der Waals contacts with nearby residues Asn279 and Phe573, while may also show a possible covalent bonding interaction between the sulfur atom of 6TG and the thiol group of residue Cys276 (Fig. 2B). Unlike 6TG, 6MP lacks an amide group (Table 1). This may explain the ineffectiveness of 6MP against USP2, as there is a hydrogen-bonding interaction between the amide of 6TG and the side-chain oxygen atom of the residue Gln283. Overall, the ternary complex structure of USP2-Ub-6TG suggests the existence of an enzyme-substrate-inhibitor (ESI) complex and confirms the noncompetitive mechanism of USP2 inhibition by 6TG.

### Structural comparison of human USP2-Ub in complex with or without 6TG

**Figure 3:**
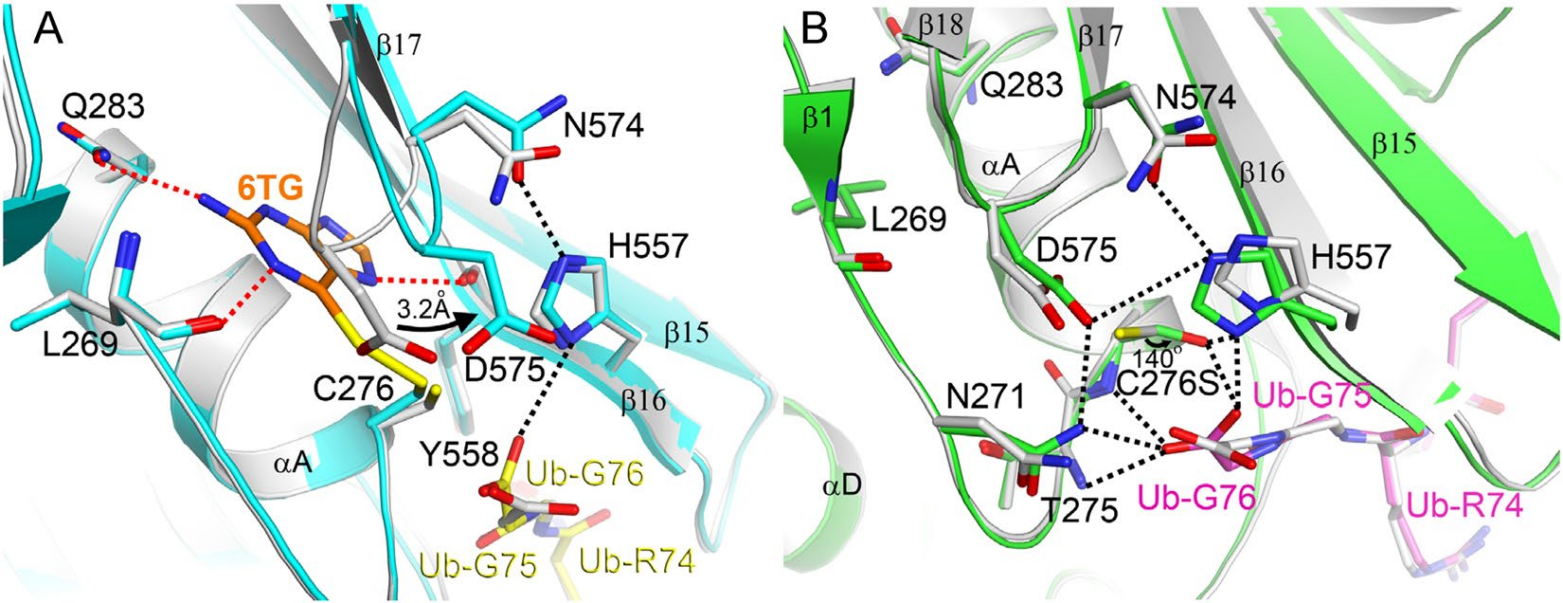
Comparison with other structures of human USP2. Overlay of the active site of human USP2-Ub complex (grey; PDB code: 2hd5) with that of USP2-Ub-6TG complex (USP2: cyan; Ub: yellow; 6TG: orange) (**A**) or that of USP2 C276S mutant (green) in complex with Ub (magenta) (**B**). The dashed lines show hydrophilic interactions. The arrow in panel (**A**) indicates the movement of residue Asp575, while that in panel (**B**) shows the side-chain movement of residue 276, which has been mutated from cysteine to serine.

Because of the partial occupancy of 6TG in the complex structure, conformational change due to ligand binding can be used to further evaluate the binding effect. Here we compare the active site of USP2 in the presence or absence of 6TG (Fig. 3A). Interestingly, binding of 6TG leads to movement of the β17–β18 loop (residues 574–577), resulting in a 3.2 Å shift of the residue Asp575 toward His557. Previous studies suggested that the absolutely conserved residue Asp575 in the USPs plays a dual role in oxyanion hole formation and maintaining the correct alignment and protonation of His557 for catalytic competency, although there is no direct interaction between them in the crystal structure of USP2-Ub complex^23,24^. Accordingly, the movement of residue Asp575 following 6TG binding also results in the inhibition of USP2. Besides, in the present structure, the carboxyl group of Ub-Gly76 has polar interaction with residue His557 (Fig. 3A). This difference may be due to the change of the free thiol group of Cys276 to disulfide, which is less negative and then has less electrostatic repulsion with the carboxyl group of Ub-Gly76.

### Structural comparison of human USP2-Ub and USP2 C276S-Ub

In an earlier study, the structure of wild-type USP2 in complex with Ub showed a misorientation of the thiol group of Cys276 due to electrostatic repulsion between the residue and the carboxyl group of Ub-Gly76^23^. To resolve this, in the present study, the crystal structure of the USP2 C276S mutant in complex with Ub was determined at 1.24 Å resolution (Table 2). Indeed, the hydroxyl group of Ser276 shows a 140° orientation change compared with the thiol group of Cys276 (Fig. 3B). This change results in the rescue of the hydrogen-bonding interaction between Ser276 and His557 and the reconnection of the catalytic triad even in the presence of Ub. Interestingly, but not surprisingly, the USP2 C276S mutant still maintains 10% deubiquitinating activity (Fig. 1B). This indicates that the C276S mutant is less active but not fully inactive. Furthermore, in the presence of 40 μM 6TG, the DUB activity of the USP2 C276S mutant decreases to 30%, suggesting that 6TG can still inhibit the enzyme without the disulfide-bonding interaction. Compared with those of wild-type USP2, the residues interacting with 6TG, such as Leu269, Gln283 and Tyr558, have no conformational change in C276S mutant, indicating the existence of 6TG binding pocket (Fig. 3B). The movement of residue Asp575 following 6TG binding may also result in the inhibition of C276S mutant. Moreover, in the present structure, one oxygen atom of the carboxyl group of Ub-Gly76 is located in the oxyanion hole consisting of the three amides from Asn271, Thr275 and Ser276 (Fig. 3B). Previous studies indicated that mutation of residue Asn271 (N271A) has no influence on kinetic parameters such as k_cat_ and K_M_^24^. This indicates that the two main-chain amides from residues 275 and 276 are enough to stabilize oxyanion formation during catalysis. On the other hand, different to the USP2-Ub complex, USP2 C276S-Ub complex shows electrostatic interaction between residues Asp575 and His557 (Fig. 3B). It suggests that Asp575 may directly influence the protonation of His557, not via the interaction between Asn271 and Asp575^24^. Overall, the present structure provides a foundation for more understanding the molecular basis of USP2 catalysis.

**Table 2:** X-ray diffraction data collection and refinement statistics.

	Human USP2-Ub-6TG complex	Human USP2 C276S mutant-Ub complex
Data Collection		
Space group	*C*2	*C2*
Cell dimensions		
*a*, *b*, *c* (Å)	103.0, 54.7, 72.7	102.5, 54.0, 74.8
β (°)	107.5	107.8
Resolution^a^ (Å)	30–1.80 (1.86–1.80)	30–1.24 (1.28–1.24)
*R*_merge_^b^ (%)	5.1 (46.6)	3.4 (24.1)
*I*/*σI*	19.9 (2.4)	33.0 (5.7)
Completeness (%)	97.9 (80.1)	98.5 (96.9)
Redundancy	3.7 (3.5)	3.6 (3.4)
Refinement		
Number of reflections	33,397 (4,726)	102,800 (14,652)
*R* factor^c^ (%)	17.9 (26.7)	14.8 (15.9)
Free *R* factor^d^ (%)	23.8 (31.0)	16.4 (18.2)
Number of atoms	3,568	3,761
Protein	3,285	3,404
Ligand/ion	11/11	0/1
Water	261	356
*B*-factors (Å^2^)		
Protein	38.9	14.7
Ligand/ion	65.2 (40.7)^e^/57.9	−/13.7
Water	50.9	23.9
rmsd		
Bond length (Å)	0.012	0.007
Bond angles (°)	1.5	1.2
Ramachandran analysis (%)		
Favored	91.6	92.6
Allowed	8.4	7.5

^a^The numbers in parentheses are for the highest-resolution shell.

^b^${R}_{merge}=\sum _{h}\sum _{i}|{I}_{hi}-\langle {I}_{h}\rangle |/\sum _{h}\sum _{i}{I}_{hi}$, where *I*_*hi*_ is the integrated intensity of a given reflection and $\langle {I}_{h}\rangle$ is the mean intensity of multiple corresponding symmetry-related reflections.

^c^$R=\sum _{h}|{F}_{h}^{o}-{F}_{h}^{c}|/\sum _{h}{F}_{h}^{o}$, where ${F}_{h}^{o}$ and ${F}_{h}^{c}$ are the observed and calculated structure factors, respectively.

^d^Free *R* is *R* calculated using a random 5% of data excluded from the refinement.

^e^The value in parentheses is calculated at 50% occupancy.

### Slow-binding inhibition of USP2 by 6TG

**Figure 4:**
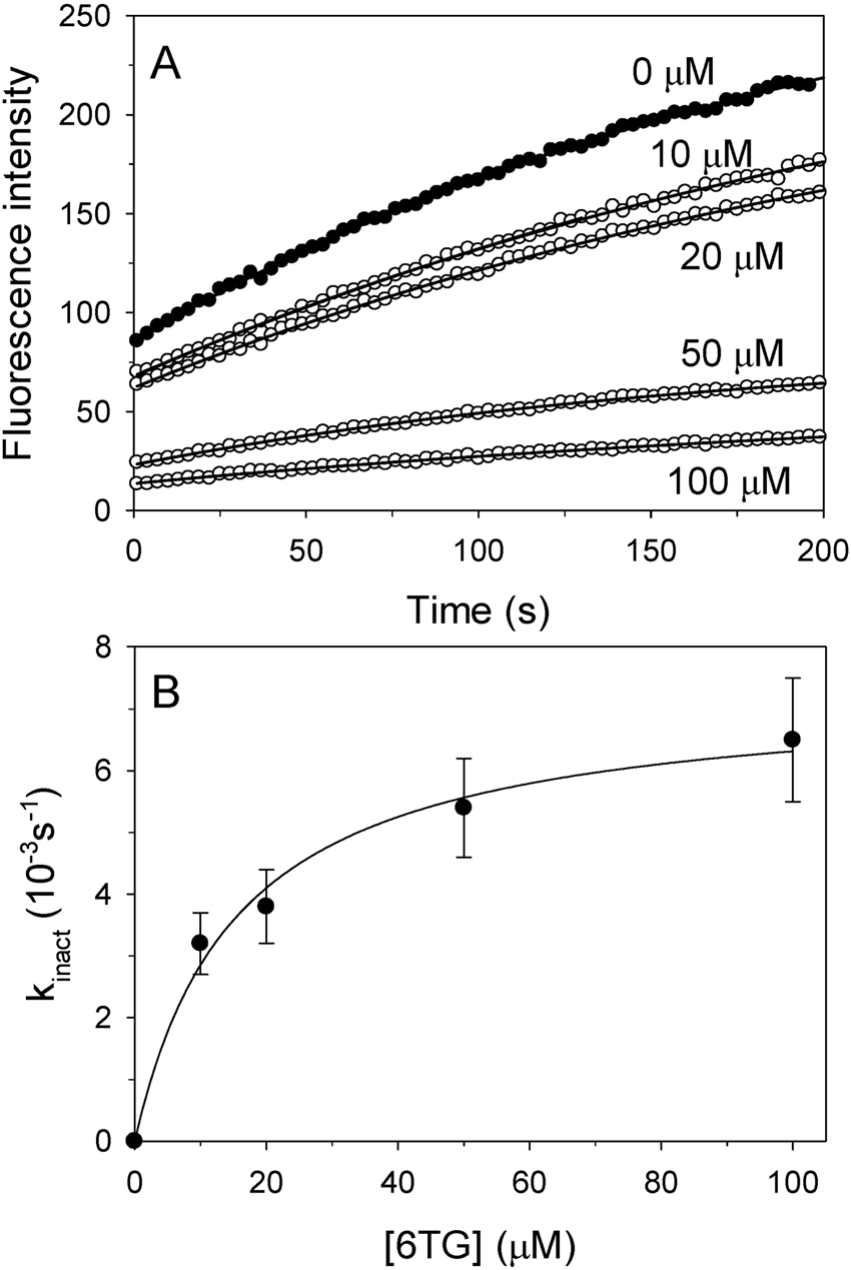
Time-dependent inactivation of USP2 by 6TG. (**A**) Different concentrations of 6TG (0 μM, closed circles; 10–100 μM, open circles) were incubated with USP2 and enzyme activity was measured for 200 s. Across all trials, Ub-AFC concentration was held at 0.5 μM and USP2 concentration was held at 0.2 μM. The solid lines show the best fit results when the data was fitted to the slow-binding equation. (**B**) The observed inactivation rate constants (k_inact_) from panel A were replotted against 6TG concentrations. The solid line represents the best fit of the data to the saturation equation. The apparent K_inact_ value is shown in Table 1.

According to the ternary structure of USP2-Ub-6TG, the enzyme may have a covalent-bonding interaction with 6TG, albeit 6TG showed partial occupancy (Fig. 2B). Although rare, the disulfide-bonding formation of 6TG has been confirmed by van der Vlies *et al*. in 2012^25^. In the present study, to validate the reversibility of the enzyme inactivation, USP2 was incubated with 1 mM 6TG for 2 h followed by removal of the small molecules using a Sephadex G-25 column. This treatment led to a 95% loss of activity, suggesting irreversible inhibition of the USP2 by 6TG. The irreversible inhibition of enzyme activity indicates that 6TG may be covalently associating with the enzyme^26^. Next, the 6TG-labelled USP2 was incubated with 2 or 20 mM βME for 10 min and then measured the activity to see if any re-activation. Although not very high, there is 14% and 20% of the enzyme activity restored after treated by βME, respectively. The rescuing effect of reductant suggests that the modification may be due to the disulfide bonding interaction between the enzyme and 6TG. Nevertheless, the above observations suggest that 6TG may be a “slow-binding” inhibitor^26^. Here we measured the time-resolved emission for 200 s under various concentrations of 6TG and found that the emission curve was curved downward, indicating a slow-binding mechanism (Fig. 4A). Different k_inact_ at various concentrations of 6TG were determined by fitting the data to Eq. 2 and then plotted versus those various concentrations of 6TG (Fig. 4B). The saturation pattern and irreversible inhibition of enzyme activity suggests that slow binding may be due to affinity labeling, in accordance with Copland’s models^26^. Following this, fitting the data to the saturation equation, we obtained a K_inact_ of 15.6 μM (Fig. 4B and Table 2). This value is even lower than K_is_, indicating that 6TG may undergo a covalent-bonding interaction with USP2 very soon after binding.

## Conclusion

Here we found that 6TG can noncompetitively inhibit human USP2. It also shows a slow-binding inhibitory effect against the same enzyme. The kinetic and catalytic mechanism was further confirmed by X-ray crystallography. The ternary complex structure of USP2-Ub-6TG can be interpreted as an enzyme-substrate-inhibitor complex, suggesting allosteric binding of inhibitor and substrate on the same enzyme. The binding of 6TG also leads to the movement of Asp575, an absolutely conserved residue playing an essential role in catalysis. This further explains why the binding of 6TG can inhibit USP2. Finally, the irreversible inhibition of the enzyme by the inhibitor and the slow-binding inhibition via affinity labeling suggested a possible covalent bonding interaction between 6TG and the residue Cys276 of USP2. Our findings once again revealed the broad spectrum of activity of 6TG and provides biochemical and structural evidence for evaluating the possible application of 6TG on the clinical or combinational treatment against those USP2-upregulated cancers.

## Materials and Methods

### Plasmid construction

The cDNA of human USP2 (Gene ID: 9099) catalytic domain (residues 258 to 605) was amplified by polymerase chain reaction with the primers 5′-GGCGCTAGCATGAATTCTAAGAGTGCCCAG-3′ and 5′-GGCCTCGAGCATTCGGGAGGGCGGGC-3′, using pOTB7-USP2 (MGC premier cDNA clone ID: BC002854) as a template. The PCR product was digested by *Nhe*I-*Xho*I and then inserted into the vector pET-24d with a 6x-His tag at the C-terminus (Novagen). The primer sequences for site-directed mutagenesis of the USP2 C276S mutant were 5′-CGAAACCTTGGGAACACGAGCTTCATGAACTCAATTCTG-3′ and 5′-CAGAATTGAGTTCATGAAGCTCGTGTTCCCAAGGTTTCG-3′. The reading frames of the above plasmids were verified by DNA sequencing.

### Expression and purification of human USP2

In the present study, we used the catalytic domain (residues 258–605) since the full-length USP2 has not been obtained despite our effort to express the full-length protein by sumo-fusion strategy. The cloned plasmid was transformed into *Escherichia coli* Rosetta (DE3) cells (Novagen). Cultures of 0.8 l LB medium with 25 μg/ml kanamycin and 17 μg/ml chloramphenicol were grown at 37 °C for 4 h and then incubated at 20 °C for 20 h after adding 0.4 mM isopropyl-β-_D_-thiogalactopyranoside. After centrifugation, the cell pellets were resuspended in lysis buffer (20 mM Tris, pH 8.5, 250 mM NaCl, 5% glycerol, 0.2% Triton X-100 and 2 mM β-mercaptoethanol (βME)), sonicated and then centrifuged at 12,000 *g* at 4 °C for 20 min to remove the insoluble lysate. The supernatant was mixed with 1 ml Ni-NTA agarose (Qiagen) at 4 °C for 1 h, washed with buffer containing 20 mM Tris, pH 8.5, 250 mM NaCl, 8 mM imidazole and 2 mM βME and then eluted with buffer containing 20 mM Tris, pH 8.5, 30 mM NaCl, 150 mM imidazole and 2 mM βME. Next, the protein was loaded onto an S-100 gel-filtration column (GE Healthcare) using running buffer (15 mM sodium phosphate, pH 6.0, 100 mM NaCl and 2 mM dithiothreitol). The purity of the collected fraction was checked by SDS-PAGE and the protein was concentrated using an Amicon Ultra-4 30 kDa filter (Millipore). The final purified protein at 15 mg/ml with 5% glycerol was flash-frozen with liquid nitrogen and stored at −80 °C. The typical yield of protein was 15 mg for wild-type USP2 and 30 mg for C276S mutant per liter of cell culture.

### Deubiquitination assays and steady-state kinetic analyses

Deubiquitination assays were performed in 20 mM phosphate buffer (pH 7.6) at 30 °C. The concentration of the fluorogenic substrate ubiquitin-7-amino-4-trifluoromethylcoumarin (Ub-AFC; Boston Biochem) was varied while the concentration of USP2 catalytic domain or its C276S mutant was held at 0.2 μM. Enzyme activity was measured by continuously monitoring the fluorescence emission increase at 485 nm upon substrate cleavage at excitation wavelength of 350 nm in a PerkinElmer LS 50B luminescence spectrometer. The increase in fluorescence was linear for the first 25 s. Kinetic parameters were obtained by fitting the initial velocities (v_i_) to the Michaelis-Menten equation. The program Sigmaplot 12 (Systat Software) was used for data analysis.

For the inhibition assays, concentrations of the substrate Ub-AFC and an inhibitor, 6-mercaptopurine (6MP) or 6TG, were varied while the concentration of the USP2 catalytic domain was held at 0.2 μM. The inhibition data for the inhibitors was found to best fit a noncompetitive inhibition model according to Eq. (1):

1 $${v}_{i}={V}_{max}[S]/((1+[I]/{K}_{is}\,)({K}_{M}+[S]))$$

in which *V*_*max*_ is the maximal initial velocity of the enzyme while *[S]* and *[I]* denote the concentrations of the substrate and inhibitor, respectively. *K*_*M*_ is the Michaelis-Menten constant for the enzyme-substrate complex and *K*_*is*_ is the inhibition constant for the enzyme-inhibitor complex.

### Slow-binding inhibition mechanism

To study slow-binding inhibition by 6TG, the USP2 catalytic domain at a concentration of 0.2 μM was incubated with different concentrations of 6TG in the presence of 0.5 μM Ub-AFC substrate. Enzyme activity was traced for 200 s and all of the progress curves showed exponential decay. The experimental data was analyzed according to the integrated rate equation (Eq. 2)^18,26^:

2 $$[P]={v}_{s}t+({v}_{i}+{v}_{s})\,(1-exp(-{k}_{inact}\,t))/{k}_{inact}$$

in which *v*_*s*_ is the steady-state velocity, *k*_*inact*_ is the apparent first-order rate constant for the interconversion between *v*_*i*_ and *v*_*s*_ and *t* is time.

### Protein crystallization

Crystals of the human USP2 catalytic domain or C276S mutant in complex with Ub (Sigma-Aldrich) were obtained by the sitting-drop vapor-diffusion method. After incubating overnight, the USP2-Ub mixture (6.6:3.3 mg/ml; molar ratio 1:2) was crystallized in a reservoir solution consisting of 24.5% PEG 3350, 0.2 M MgCl_2_ and 0.1 M Bis-Tris (pH 7.0), while the USP2 C276S-Ub mixture was crystallized in a solution of 21% PEG 3350 and 8% Tacsimate (pH 8.0). Single diamond-shaped crystals 0.2–0.3 mm in size grew at 22 °C after a week. The USP2-Ub crystals were soaked in reservoir solution supplemented with 1 mM 6TG for 4 h and then flash-cooled in liquid nitrogen.

### X-ray data collection, structure determination and refinement

X-ray diffraction data were collected at 100 K on beamlines BL13C1 and BL15A1 at the National Synchrotron Radiation Research Center, Taiwan, ROC, using an ADSC Q315r or Rayonix MX300HE CCD detector (X-ray wavelength 0.976 Å). The image data was processed and scaled with the HKL-2000 package^27^. Both kinds of crystals belong to space group C2; the cell dimensions of the USP2-Ub-6TG crystal were a = 103.0, b = 54.7, c = 72.7 Å, α = γ = 90° and β = 107.5°, while those of USP2 C276S-Ub were a = 102.5, b = 54.0, c = 74.8 Å, α = γ = 90° and β = 107.5°. The crystallographic asymmetric unit contained one USP2 catalytic domain and one Ub. The structures were solved by the molecular replacement method with Phaser^28^ using the structure of USP2-Ub (PDB code: 2hd5^23^) as the model. Structural refinement was carried out using REFMAC^29^ and the atomic model was built using Coot^30^.

### Accession codes

The final structure information has been deposited in the Protein Data Bank (PDB code: 5xu8 and 5xve for USP2-Ub-6TG and USP2 C276S-Ub, respectively).

## Electronic supplementary material

Supplementary material
