# Supplementary material for: 6-Thioguanine is a noncompetitive and slow binding inhibitor of human deubiquitinating protease USP2
Source: Sci Rep. 2018 Feb 15;8:3102. doi: 10.1038/s41598-018-21476-w (PMC5814560; doi:10.1038/s41598-018-21476-w)
Supplement: Supplementary file 1 — Supplementary material [file 41598_2018_21476_MOESM1_ESM.docx]

**Supplementary Information for**

**6-Thioguanine is a noncompetitive and slow binding inhibitor of human deubiquitinating protease USP2**

Shang-Ju Chuang^a^, Shu-Chun Cheng^b^, Hui-Chi Tang^a^, Chiao-Yin Sun^b^, and Chi-Yuan Chou^a*^

^a^Department of Life Sciences and Institute of Genome Sciences, National Yang-Ming University, Taipei 112, Taiwan

^b^Department of Nephrology, Chang-Gung Memorial Hospital, Keelung 204, Taiwan

^*^Correspondence information for Dr. Chi-Yuan Chou. Address: 155 Li-Nong St., Sec. 2, Taipei 112, Taiwan, R.O.C. Phone: +886-2-28267168. FAX: +886-2-28202449. E-mail: [cychou@ym.edu.tw](mailto:cychou@ym.edu.tw)


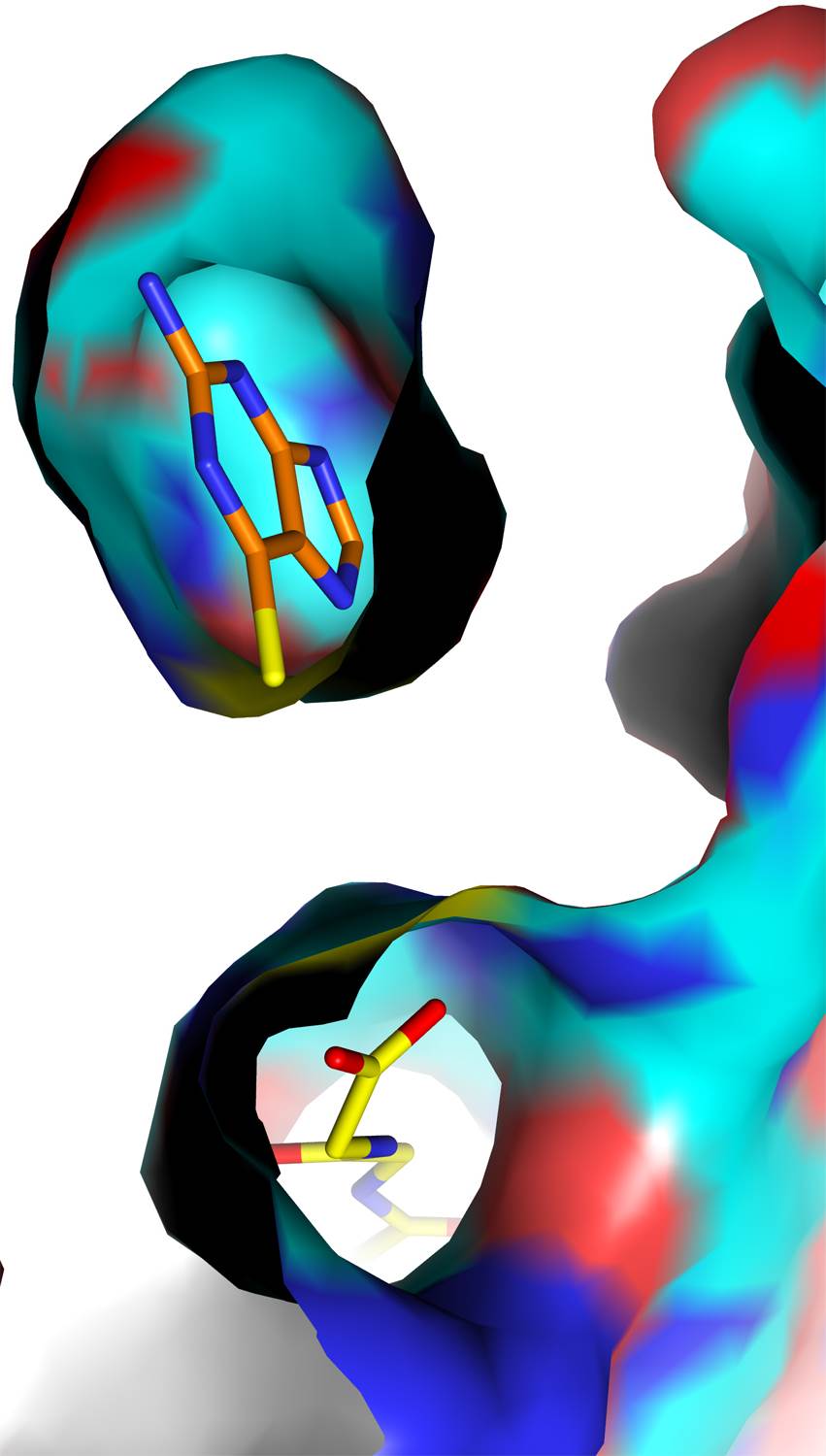

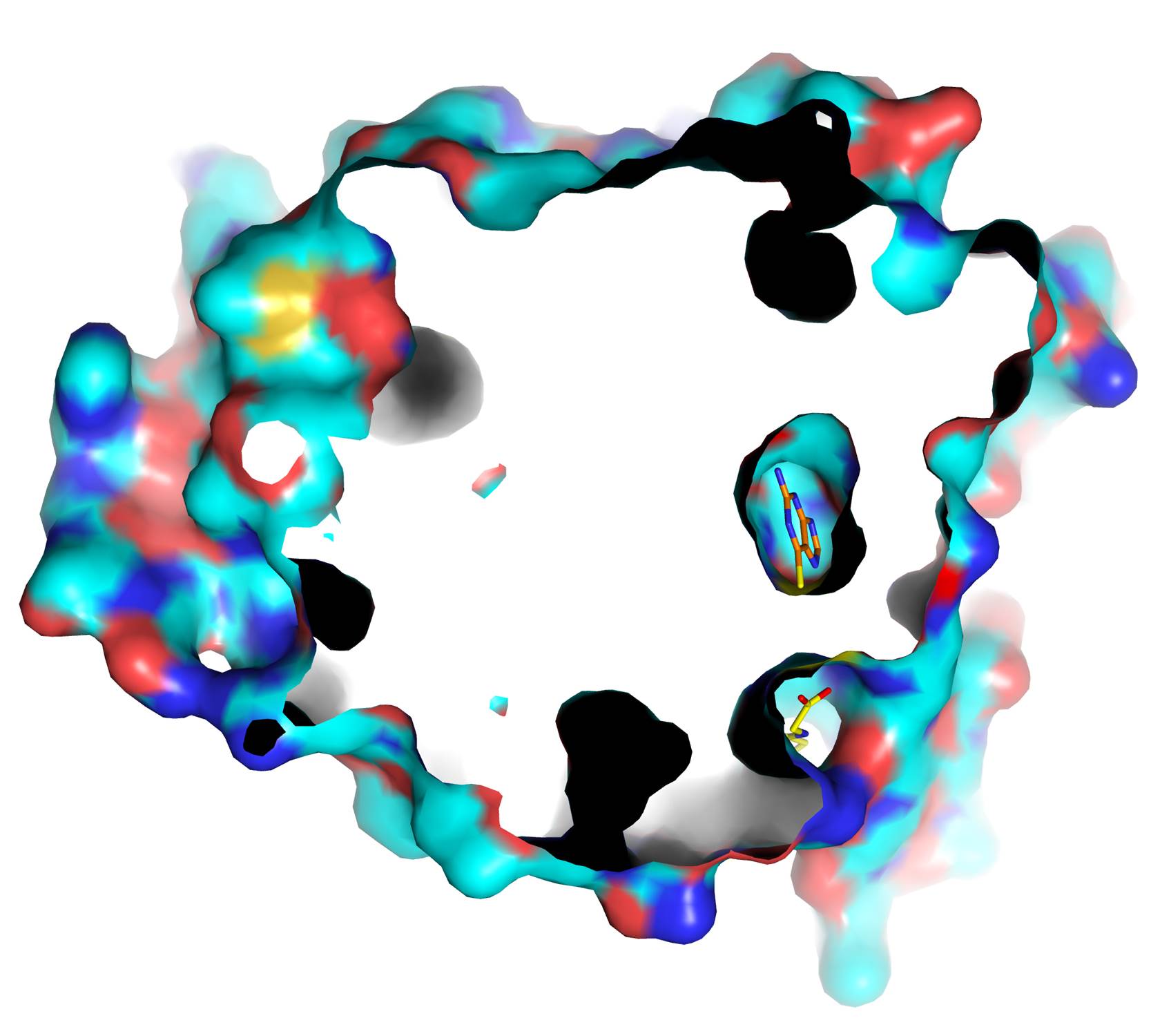


**Extended Data Fig. 1.** The molecular surface of human USP2 in complex with Ub and 6TG. Left panel shows the overall structure while the right panel shows the amplification of the active site of USP2. The 6TG (orange) and the C-terminus of Ub (yellow) are shown by sticks.
